# Authors' Reply

**DOI:** 10.1371/journal.pcbi.0020083

**Published:** 2006-07-28

**Authors:** Hung D Nguyen, Maki Yoshihama, Naoya Kenmochi

## Authors' Reply

We thank Csűrös for his thoughtful remarks about our recent paper in *PLoS Computational Biology* [1,2]. Although both our method [2] and that of Csűrös [3] (the latter was published when the former was under review) assume the same model of intron evolution, and the results obtained by both methods for the dataset in [4] are similar, the implementation details are quite different. Csűrös [3] used a trial-and-error procedure for guessing the number of unobserved intron sites, and used posterior probability calculation to optimize the rates of intron gain and loss. In contrast, in our method the number of unobserved intron sites and rates of intron gain and loss are inferred at the same time by maximizing a likelihood function. As pointed out by Csűrös [1], the recent result of Raible et al. in *Science* [5], which indicated that about two-thirds of Platynereis dumerilii introns are at the same positions as those in humans seems to support the results in [2] and [3]. It is likely that the evolution of introns in P. dumerilii was similar to that in humans, where two-thirds of the introns were already present in the last common ancestor and the remaining one-third was gained late after divergence from this ancestor.

Csűrös [1] commented that his algorithm is more efficient in terms of running time compared with ours. This comment is correct but with the stipulation that the number of observed sites be less than 2*^N^,* where *N* is the number of species. This condition does not hold for the dataset in [4]. In practice, however, this condition often holds for large values of *N*. In this case, some of the equations in our method [2] can be rewritten to yield the same time complexity as the method in [3].

Suppose that there are *U* observed intron sites that belong to *V* (*V* ≤ *U*) intron patterns. Denote *n_i_* (*i* = 0..*V*) to be the number of intron sites for pattern *i*. Note that *n*_0_ *,* which is the number of unobserved intron sites, is unknown. The log-likelihood function in our method (Equation 7 in [2]) can be now rewritten as:

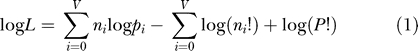
where *p_i_* is the expected probability of intron sites of pattern *i* and *P* = *U* + *n*_0_ is the total number of sites. Although the time to compute *p_i_* (Equation 5 in [2]) appears to grow exponentially with *N,* in fact it can be computed in linear time using the well-known “pruning” technique of Felsenstein [6]. For each pattern *i* and each node *x* we compute the two conditional likelihoods *L_ix_* ^(0)^ and *L_ix_* ^(1)^ for states 0 (intron absence) and 1 (intron presence), respectively, using the post-order tree traversal. After that we compute:

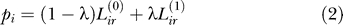
where *λ* is the probability of introns being present at the root node and *L_ir_* ^(0)^ and *L_ir_* ^(1)^ are the two conditional likelihoods of the root node for pattern *i*.

Since all the conditional likelihoods for every node are now already known, the expected counts of intron gain and loss for each intron pattern *i* along each branch *k*, *g_ik_,* and *l_ik_,* as well as the expected intron counts at each node *h, o_ih_,* can also be computed in linear time with *N* using the pruning technique, but this time with the pre-order tree traversal. Finally, Equations 9 and 10 in [2] can be rewritten as:

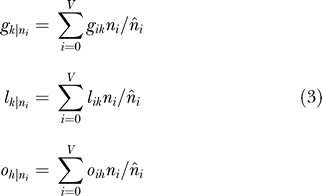
where 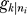 and 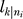 are, respectively, the conditional expected counts of intron gain and loss for each branch *k* given the data; 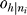 is the conditional expected intron count for each node *h* given the data; and 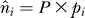 is the expected number of sites for each intron pattern *i*. In this way, our algorithm also has a linear time with *N* and *V*. In fact, the code that was released together with our paper [2] has already been implemented using the pruning technique.

Csűrös [1] also commented that our Proposition 1 echoes the Pulley Principle of Felsenstein [6] for ambiguous root placement. The Pulley Principle, however, applies to only reversible Markov processes whereas our model of intron evolution is an irreversible one. Concerning our Proposition 2, Csűrös [1] is correct to comment that our method for finding the most biologically meaningful solution, which is based on the variance of intron gains and losses, is less efficient. Since our algorithm is initialized with very small rates of intron gain and loss, the algorithm almost always converges to the most biologically meaningful solution (although there is no direct proof for this). Thus, the step of finding the most biologically meaningful solution was added to make the algorithm more rigorous, and may be removed in practice.

Although the use of Equation 1 in [1] will lead to a unique solution with the method of Csűrös, the method may not always find a solution with the maximum likelihood. This happens when no solution among the 2*^N^* ^−2^ optimal solutions (see our Proposition 2 in [2]) satisfies the condition *p_e_*(0 → 1) + *p_e_*(1 → 0) < 1 in [1] (or *α_k_* + *β_k_* < 1 in our terms, where *α_k_* and *β_k_* are the probabilities of intron gain and loss along branch *k*, respectively [2]). Let us consider the following example using Figure S3 in [2]. Suppose that *C* and *D* are external nodes (i.e., they present the observed data) and *o_C_* = 420 and *o_D_* = 160, where *o_X_* shows the number of introns at node *X*. We suppose further that *P* = 1,000 and that one optimal solution for node *B* has the following parameters: *o_B_* = 400, *g_y_* = 60, *l_y_* = 40, *g_z_* = 120, and *l_z_* = 360. In this case, *α_y_* = 0.1, *β_y_* = 0.1, *α_z_* = 0.2, *β_z_* = 0.9, and *α_z_* + *β_z_* > 1. According to our Protocol S2, the other optimal solution for node *B* has the following parameters: *o_B_* = 600, *g_y_* = 360, *l_y_* = 540, *g_z_* = 40, and *l_z_* = 480. In this case, *α_y_* = 0.9, *β_y_* = 0.9, *α_z_* = 0.1, *β_z_* = 0.8, and *α_y_* + *β_y_* > 1. That is, both optimal solutions for node *B* violate the condition *α_k_* + *β_k_* < 1, and the method of Csűrös may not find an optimal solution in this case. Therefore, to always find the most biologically meaningful solution in linear time using our method, we propose to use a new definition: the most biologically meaningful solution is the one that has the least total number of intron gains and losses. Now we can compute the most biologically meaningful solution from any arbitrary optimal solutions by using the post-order tree traversal, and for each internal node *B* choose the optimal solution for which the sum *g_y_* + *l_y_* + *g_z_* + *l_z_* is smaller (i.e., the case *o_B_* = 400 in the above example). The algorithm clearly has a linear time with *N*.

Csűrös [3] stated that it was not possible to estimate *n*_0_ (i.e., the number of unobserved intron sites) by means of likelihood. Therefore, a trial-and-error procedure, which basically tries all possible values for *n*_0_, was used. Our method, however, suggests that *n*_0_ can be optimized by means of likelihood. The problem may be that the method in [3] attempts to maximize a log-likelihood function similar to the one in Equation 1 (in this reply) but without the last two terms. When *n*_0_ is invariant, these two terms are constant and can be omitted. However, they cannot be omitted when inferring intron evolution (where *n*_0_ is unknown) if we want to optimize *n*_0_ by means of likelihood. One advantage of using our log-likelihood function is that we can use conventional methods (such as the Brent algorithm) for optimizing *n*_0_, which are more efficient than the trial-and-error method employed in [3]. Another advantage of using our function is that different trees can be compared on the basis of their likelihoods [2]. The method in [3] does not allow such a comparison.

Our intent when comparing the number of possible patterns with the number of intron sites in the dataset in [7] was to show that the dataset may be insufficiently large for a valid inference, i.e., other methods such as maximum parsimony may perform better in this case. It was not our intent to claim that the sample data must grow proportionally with the number of possible patterns for a statistical inference to be valid. Our recent simulations (unpublished data) seem to support our speculations about the dataset in [7]. 
